# Association between Caspase-1, TNF-α Salivary Level and Their Diagnostic Potential to Discriminate Periodontitis from Healthy Control

**DOI:** 10.3290/j.ohpd.b3904349

**Published:** 2023-02-16

**Authors:** Athraa A. Mahmood, Raghad Fadhil Abbas

**Affiliations:** a Assistant Professor, Oral Surgery and Periodontology Department, College of Dentistry, Mustansiriyah University, Baghdad, Iraq.; b Assistant Professor, Department of Periodontology, College of Dentistry, University of Baghdad, Baghdad, Iraq.

**Keywords:** caspase-1, periodontitis, saliva, TNF-α

## Abstract

**Purpose:**

Periodontitis is associated with caspase and proinflammatory mediators, such as caspase-1 and tumor necrosis factor-alpha (TNF-α). The aim of this study was to evaluate the salivary levels of caspase-1 and TNF-α and determine their accuracy in differentiating periodontitis patients from individuals with a healthy periodontium.

**Materials and Methods:**

This case-control study enrolled 90 subjects, aged 30 to 55, attending the Department of Periodontics at Baghdad’s outpatient clinic. Patients were initially screened to evaluate their eligibility for recruitment. After applying inclusion/exclusion criteria, subjects with a healthy periodontium were included in group 1 (controls), while subjects with periodontitis were included in group 2 (patients). The salivary levels of caspase-1 and TNF-α in participants’ unstimulated saliva were measured using an enzyme-linked immunosorbent assay (ELISA). Then the periodontal status was determined using the following indices: full-mouth plaque, full-mouth bleeding on probing, probing pocket depth, clinical attachment level, and gingival recession.

**Results:**

TNF-α and caspase-1 salivary levels were higher in periodontitis patients than in healthy controls and were positively correlated with all clinical parameters. A positive significant correlation between TNF-α and caspase-1 salivary levels was noticed. For differentiating periodontal health and periodontitis, the area under the curve (AUC) values of TNF-α and caspase-1 were 0.978 and 0.998, while the proposed cut-off points were 128.163 pg/ml and 1.626 ng/ml, respectively.

**Conclusion:**

The present findings supported a previous discovery that periodontitis patients have significantly higher levels of salivary TNF-α. In addition, there was a positive correlation between the salivary levels of TNF-α and caspase-1. Furthermore, caspase-1 and TNF-α showed high sensitivity and specificity in the diagnosis of periodontitis, as well as distinguishing periodontitis from periodontal health.

Periodontitis is a destructive inﬂammatory process believed to have a complex multifactorial aetiopathogenesis. It is caused by an imbalance between the host’s immune response and the number of pathogenic microbes in the subgingival environment, which damages tooth-supporting tissues in people around the world.^5,6,14,16,32^

Caspases, a family of intracellular protease enzymes that are key mediators of apoptosis or programmed cell death during inflammation, can be classfied into three types, depending on function.^22^ Initiator caspases, such as caspase-2, -8, and -9, initiate the apoptosis signal, while the eﬀector caspases, such as caspase-3, -6, and -7, break down different cellular substrates to cause morphological changes. Inflammatory caspases, such as caspase-1, -4, -5, and -11 to -14, are involved in inflammatory cytokine signaling and activate nuclear factor-kappa β (NF-κβ).^15,18,31^

The gingivae and dental alveolar bone are involved in the inflammatory process, of which NF-κβ is a crucial regulator.^38^ Activation of NF-κβ can induce activation and stimulation of a variety of biomolecular mediators, including TNF-α, interleukin-6 (IL-6), and IL-1β. Thus, TNF-α secretion during periodontitis is dependent on inflammatory caspase activation following recognition of a pathogenic or harmful effect.^1,7,12,20,25^

TNF-α, a major proinflammatory mediator, is crucial in periodontitis-mediated additional periodontal tissue loss and bone resorption, because it stimulates activation, proliferation, and differentiation of osteoclasts, which results in bone loss.^2,13^ Caspase activation orchestrates a localised chronic inflammatory response, and is necessary for TNF-α expression in periodontitis.^21,26^ Both healthy and periodontitis-affected saliva can be tested for caspase activation and TNF-α expression. As a result of caspase activation, the higher concentration of TNF-α seen in periodontitis closely correlates with the immune response and tissue destruction.^24,34^

In this study, the salivary levels of caspase-1 and TNF-α were measured in a sample of Iraqi periodontitis patients and healthy-periodontium controls using an ELISA technique, and were then correlated with clinical parameters (full-mouth PI, full-mouth BOP, PPD, CAL, and GR indices) to diagnose periodontitis and to distinguish periodontitis patients from healthy controls.

## Materials and Methods

The present investigation was designed as an observational case-control study conducted in Baghdad, Irak, from March 2022 to August 2022. The ethics committee of the University of Baghdad’s College of Dentistry approved the study.

**Fig 1:**
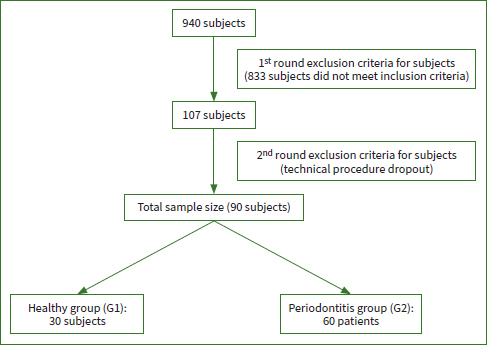
Flowchart of patient selection and sample size.

The recruitment of participants is depicted in Fig 1. Before enrollment in the study, the medical and dental history of each patient was recorded using a questionnaire. This included items about the subject’s name, age, sex, medication used, smoking or alcohol consumption, the full medical history, as well as the history of the previous periodontal treatment. In addition, each patient was asked to sign a consent form, which included written information fully explaining the nature and aims of the study. Then, salivary samples were collected from each subject followed by a clinical examination.

All participants (37 females and 53 males) recruited for this study were systemically healthy, within the normal weight range according to the body-mass index (BMI 18.5–29.9),^19^ had a minimum of 20 teeth, were cooperative and willing to sign an informed consent form, were nonsmokers, had not undergone periodontal treatment in the last 6 months, had not been on any medications in the last 3 months, had no recent acute illness symptoms (e.g. COVID-19), or any oral lesion that was not related to periodontitis.

The study’s healthy control group (n = 30 subjects) had a healthy, intact periodontium, with BOP < 10%, PPD ≤ 3 mm, and no CAL.^3^ The periodontitis patient group (n = 60) had detectable interdental CAL at ≥ 2 non-adjacent teeth, or ≥ 3 mm CAL on the buccal (facial) or lingual/palatal aspects, associated with PPD > 3 mm at ≥ 2 teeth.^35^ Furthermore, all patients had to have a classification of generalised, stage 1 to 4, grade A to C, unstable (PPD ≥ 4 mm with BOP or PPD >5 mm with or without BOP) periodontitis, with no risk factors, eg, diabetes mellitus (DM) and/or smoking.^35^

Wisdom teeth were excluded from the periodontal examination. The clinical parameters were measured for all existing teeth by the same examiner, which included full-mouth PI,^29^ full-mouth BOP, PPD, CAL, and GR for all patients, using a Michigan O periodontal probe (Hu-Friedy; Chicago, IL, USA).

The examined subjects were instructed to not eat or drink anything except water for at least 60 min before the samples were collected. The individuals properly cleaned their mouths with drinking water for 15 s to eliminate any food particles, bacteria, and desquamated epithelium, then waited 2 min for water clearance before the sample was collected. Salivary samples were collected from study subjects between 9 and 12 AM, using a standardised, passive saliva-drooling method for the collection of whole saliva.

The collected samples were placed in a small cooler for 30 min, and were then centrifuged at 3000 rpm for 15 min to separate the cellular debris from the salivary supernatants. Then the saliva samples were frozen at -24⁰C within 4 h of collection until assayed and analysed by ELISA. This was done to prevent bacterial growth and minimise the loss of biomarkers in the sample. The subject number previously noted on the case sheet was written on the label of the tube, along with the time and date of sample collection.

The accuracy, validity, and reproducibility of the periodontal parameter measurements were maximised by inter- and intra-examiner calibration on 5 patients until an agreement level of more than 75% was reached.

For categorical variables (PI and BOP), the targeted level was a Kappa coefficient ≥ 75%. For continuous variables (PPD, CAL, and GR), the level of agreement rounded to the nearest millimeter should be > 0.9, as determined by the interclass coefficient assay.

### Statistical Analysis

All statistical analysis of the data was performed and processed using SPSS for Windows, version 28 (IBM; Armonk, NY, USA). For continuous data, the mean, standard deviation (SD), and median were used, while frequency (number) and percentage were used for categorical variables.

The Shapiro-Wilk test was used to determine the normality of data distribution. Mann-Whitney U-tests were used to determine statistically significant differences between the groups studied and test the null hypothesis. In addition, Spearman’s correlation coefficient determined the statistically significant correlations between different parameters and variables. Categorical variables were analysed with the Χ^2^ test, and p-values < 0.05 were defined as statistically significant. The area under the curve (AUC) and the receiver operating characteristic (ROC) curve were used to assess the diagnostic efficacy of biomarkers.

## Results

Table 1 shows the demographic and periodontal parameters of the 90 participants (cases and controls). The mean age of patients was 41.6 ± 8.3 years, and that of the control participants was 38.3 ± 6.4 years. The proportion of males to females in the patient group was 35/25, and 18/12 in the control group. There were statistically non-significant differences in age and gender between the two study groups (p > 0.05).

**Table 1:** Demographic and clinical variables by group

Parameters		Healthy group (control)	Periodontitis group	p-value
Age	Mean ± SD	38.33 ± 6.42	41.58 ± 8.27	0.083 NS
	Median	36	40.50	
Sex	Male	18 (60.0%)	35 (58.3%)	0.880 NS
	Female	12 (40.0%)	25 (41.7%)	
PI	Mean ± SD	0.16 ± 0.10	0.63 ± 0.22	0.000^***^
	Median	0.13	0.68	
BOP	Mean ± SD	0.06 ± 0.03	0.47 ± 0.18	0.000^***^
	Median	0.06	0.45	
PPD (mm)	Mean ± SD	0.000 ± 0.000	4.54 ± 0.62	0.000^***^
	Median	0.000	4.33	
CAL (mm)	Mean ± SD	0.000 ± 0.000	3.46 ± 1.00	0.000^***^
	Median	0.000	3.300	
GR (mm)	Mean ± SD	0.000 ± 0.000	2.25 ± 1.16	0.000^***^
	Median	0.000	2.28	

p ≥ 0.05, non-significant (NS); p ≤ 0.001, significant***. SD: standard deviation; PI: full-mouth plaque index; BOP: full-mouth bleeding on probing; PPD: probing pocket depth; CAL: clinical attachment loss; GR: gingival recession.

Furthermore, the current results revealed a statistically significant increase in mean levels of PI, BOP, PPD, CAL, and GR among periodontitis patients (0.63 ± 0.22, 0.47 ± 0.18, 4.54 ± 0.62, 3.46 ± 1.00 and 2.25 ± 1.16, respectively) as compared to control group.

In addition, Table 2 demonstrates the statistically significant increase (p < 0.000) found in the median salivary levels of each salivary biomarker, i.e. TNF-α and caspase-1, in the periodontitis group (151.036 pg/ml and 3.022 ng/ml, resp) when compared to the control group (82.768 pg/ml and 1.298 ng/ml, resp).

**Table 2:** Salivary level of caspase-1 and TNF-α by group

Parameters		Healthy group	Periodontitis group	p-value
TNF-α (pg/ml)	Mean ± SD	85.39 ± 21.01	156.76 ± 27.34	0.000^***^
	Median	82.77	151.04	
	Range	58.44 – 158.41	122.91 – 281.85	
Caspase-1 (ng/ml)	Mean ± SD	1.31 ± 0.17	3.22 ±1.04	0.000^***^
	Median	1.30	3.02	
	Range	0.94 – 1.68	1.55 – 6.48	

p ≤ 0.001, significant ***; SD: standard deviation.

As shown in Table 3, using Spearman’s rank correlation coefficient (r), this study discovered statistically significant correlations (p < 0.000) between salivary levels of caspase-1/TNF-α and clinical parameters (PI, BOP, PPD, CAL, and GR). Furthermore, there were statistically significant correlations between salivary biomarkers and age. TNF-α and caspase-1, on the other hand, were not significantly correlated with sex.

**Table 3:** Correlation between salivary biomarkers, clinical parameters, and demographic data

Parameters	Salivary TNF-α		Salivary caspase-1	
	r	p-value	r	p-value
PI	0.641	0.000^**^	0.539	0.000^***^
BOP	0.726	0.000^**^	0.721	0.000^***^
PPD	0.772	0.000^**^	0.783	0.000^***^
CAL	0.768	0.000^**^	0.793	0.000^***^
GR	0.706	0.000^**^	0.703	0.000^***^
Age	0.213	0.044*	0.329	0.002^**^
Sex	0.118	0.269 NS	0.066	0.533 NS
	Salivary TNF-α			
	r	p-value		
Salivary caspase-1	0.714	0.000^**^		

r: Spearman’s rank correlation; NS: non-significant at p ≥ 0.05; statistically significant at ** p≤ 0.01, *** p ≤ 0.001: PI: full-mouth plaque index; BOP: full-mouth bleeding on probing; PPD: probing pocket depth; CAL: clinical attachment loss; GR: gingival recession.

**Fig 2:**
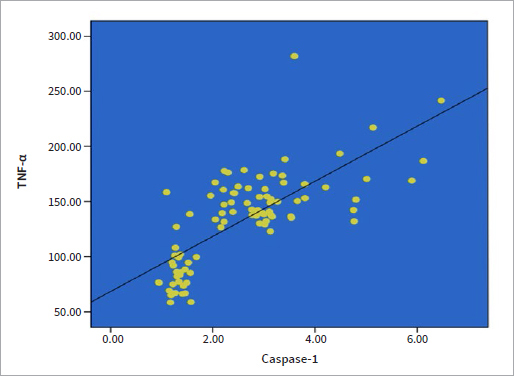
Linear regression correlation for salivary TNF-α and caspase-1.

Surprisingly, there was a statistically significant correlation (r = 0.714, p < 0.000) between caspase-1 and TNF-α salivary levels (Table 3). Linear regression was performed to describe this correlation between the two cytokines (Fig 2).

**Fig 3:**
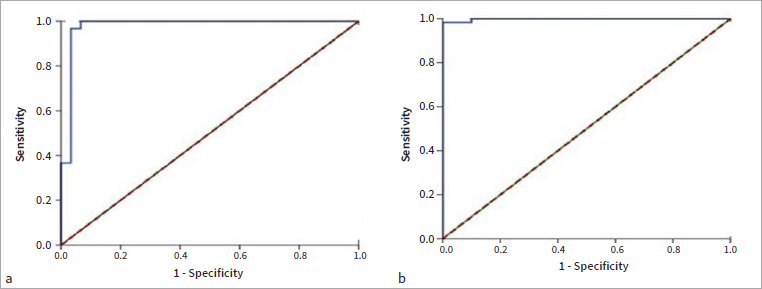
ROC curves of salivary biomarkers for healthy control vs periodontitis. (A) TNF-α; (B) caspase-1.

Based on the results of the ROC curve, which was used for differentiating periodontal health from periodontitis, the results of AUC for TNF-α and caspase-1 were 0.978 and 0.998, respectively. The proposed cut-off points were 128.163 pg/ml and 1.626 ng/ml for TNF-α and caspase-1, respectively (Fig 3 and Table 4).

**Table 4:** Sensitivity, specificity, and cut-off points of salivary biomarkers

Biomarker	Sensitivity	Specificity	AUC	95% CI	Cut-off point
Healthy vs periodontitis					
TNF-α (pg/ml)	0.967	0.967	0.978	0.937–1.000	128.163
Caspase-1 (ng/ml)	0.983	0.967	0.998	0.994–1.000	1.626

## Discussion

The IL-1 converting enzyme, caspase-1, is implicated in the necrotic, inflammatory death of host cells.^27^ Salivary caspase-1 levels in this study were statistically significantly higher in the periodontitis group than in the the group with a healthy periodontium. This suggests that the level of periodontal tissue damage and the rise in salivary caspase-1 are related.

The rise in caspase-1 concentration follows the activation of different cells (epithelial cells, leukocytes, fibroblasts of periodontal ligament, and osteoblasts) by the inflammatory stimulus to produce pro-inflammatory factors such as IL-1β and TNF-α. These are thought to be crucial in the destruction of connective tissue and bone, as well as in periodontal attachment loss in periodontitis, as a result of the activation of osteoclasts and matrix metalloproteases (MMP).^4,8,23,33,36^

No research has looked into the relationship between caspase-1 and TNF-α levels and periodontitis up until this point. Furthermore, only a small number of researchers have found evidence to support the link between TNF-α and periodontitis,^10,24^ and even fewer have found evidence to support the association.^30,34^ Therefore, using the new 2017 classification system for periodontal disease, the purpose of this study was to compare the levels of TNF-α and caspase-1 in the saliva of periodontitis patients vs periodontally healthy subjects .

In this cross-sectional investigation, TNF-α levels in the saliva of patients and periodontally healthy subjects were calculated and compared to caspase-1 levels. TNF-α is a pro-inflammatory cytokine that influences the activation of inflammatory leukocytes, change of vascular permeability, and stimulation of bone resorption.^37^

The current study found a higher level of salivary TNF-α in the patient than in the control group. This agrees with the studies by Varghese et al^37^ and Ehsan et al,^9^ who noticed that individuals with chronic periodontitis had TNF-α values substantially greater than those of control subjects.

Additionally, there was a statistically significant correlation between generalised periodontitis parameters and the presence of TNF-α (p < 0.000). These results point to a potentially harmful effect of TNF on periodontal tissues. These findings were in agreement with studies by Geng et al^10^ and Mahmood and Al-Ghurabi,^24^ who found that patients with chronic periodontitis had higher levels of this cytokine compared to people with a healthy periodontium. These studies also concluded that TNF-α and IL-6 may be used as diagnostic biomarkers for periodontitis.

In contrast, Ng et al^28^ and Rathinasamy et al^30^ found no statistically significant difference in the level of salivary TNF-α between chronic periodontitis and healthy subjects (p > 0.05). The precise role of TNF-α in periodontitis is still unknown.

As we followed a new classification of periodontitis, this variability in outcomes may be due to different patient selection criteria. Here, the periodontitis patients demonstrated a higher level when compared to the periodontally healthy subjects; the difference in the results may be attributed to various potential confounders, including different age groups, differences in sampling (stimulated or unstimulated), restricted samples of study, dissimilar processing of centrifugation, storage time, storage temperature, and biomarker evaluation techniques (enzyme-linked immunohistochemistry) or variations in the analysis kits.

In addition, the Teles et al^34^ study showed results dissimilar to those of this study; they found no correlation between the levels of salivary TNF-α and periodontal parameters. They attributed their findings to the inhibition of cytokines by putative inhibitors that were present in whole saliva.

Another interesting finding in this study was the stastistically significantly positive association of salivary TNF-α with PPD, CAL, and GR. Correspondingly, previous Iraqi studies demonstrated a statistically significantly positive correlation of TNF-α levels with periodontal destruction parameters (PI, GI, PPD, and CAL), demonstrating this cytokine’s contribution to the onset of periodontal disease.^11,24^

Similarly, in samples of individuals with chronic and aggressive periodontitis, Kurtis et al^17^ found a strong association between salivary TNF-α levels and periodontal clinical indices (PPD, CAL, PI, and GI). Unlike the current results, Varghese et al^37^ reported a statistically non-significant correlation between TNF-α and periodontitis parameters. This finding may be attributed to the extensive dilution of this marker in whole saliva, which makes it unable to reflect the tiny variations in periodontal parameters.^37^

Interestingly, a strong positive correlation was found among the levels of the caspase-1, proinflammatory cytokines (TNF-α) and the periodontitis parameters in the present study, supporting the hypothesis that these biomarkers may play a considerable role in triggering processes that result in the chronic inflammation of periodontitis. Thus, selected salivary biomarkers showed high sensitivity and specificity in the diagnosis of periodontitis and distinguishing it from periodontal health at the proposed cut-off points (128.163 pg/ml of proinflammatory cytokines [TNF-α] and 1.626 ng/ml of caspase-1).

A limitation of the current study was that only systemically healthy nonsmokers were included, and the severity of periodontitis was not considered. Thus, it is suggested that future research measure the level of TNF-α and caspase-1 relative to the severity of periodontitis. In addition, other proinflammatory and anti-inflammatory cytokines need to be investigated for a possible association with caspase-1 and periodontitis. Further investigations using other types of samples, such as gingival tissues and GCF, are necessary. Nevertheless, the precise role of caspase-1 in the pathogenesis of periodontal disease remains unknown. Thus, further research into caspase-1 activity in periodontitis is required, particularly at the molecular level.

## Conclusion

Caspase-1 and TNF-α salivary levels were increased in periodontitis patients compared to the healthy controls, and were positively correlated with clinical parameters. Furthermore, the results of this study confirmed the interactive relationship between TNF-α and caspase-1 salivary levels, which may play a considerable role in triggering the processes that lead to chronic inflammation in clinical periodontitis. The salivary biomarkers caspase-1 and TNF-α showed high diagnostic accuracy in distinguishing periodontal health from periodontitis.
